# Ovarian Teratomas in Children and Adolescents: Our Own Experience and Review of Literature

**DOI:** 10.3390/children9101571

**Published:** 2022-10-18

**Authors:** Fani Gkrozou, Orestis Tsonis, Anastasia Vatopoulou, Georgia Galaziou, Minas Paschopoulos

**Affiliations:** 1 Department of Obstetrics and Gynaecology, Medical School, University of Ioannina, 45500 Ioannina, Greece; 2 Assisted Conception Unit, Guy’s and St Thomas’ NHS Foundation Trust, London SE1 9RT, UK

**Keywords:** ovarian teratomas, children, adolescents, laparoscopy, laparotomy

## Abstract

**Background**: Ovarian teratomas are the most common type of ovarian mass during childhood and adolescence. There is no clear guidance for the management of the ovarian teratomas in children and adolescents. It is necessary, however, to understand the feature of these tumours and the indications for operating on them, and to put subjective criteria for the management in elective or emergency presentations. **Methods**: Medical records of patients between the age of 12 and 20 years old that underwent surgery for ovarian teratomas at the Department of Obstetrics and Gynaecology in University Hospital of Ioannina, between January 2000 and August 2022, were reviewed. A medical database was searched between January 2000 and August 2022 with the following keywords: ovarian teratoma, paediatrics and adolescents. **Results**: We present the results of forty patients, with a mean age of 11.8 years of age. All patients had surgery, where three out of four underwent oophorectomy and only one out of four underwent ovary-sparing operation; similar results were found in the literature. **Conclusion**: Ovary-sparing treatment is more common recently compared with the past, such as MIS compared with laparotomy. Better-organised and -planned prospective multi-centre studies are necessary to gain a deeper knowledge of the physiology and prognostic factors of teratomas in children.

## 1. Introduction

Ovarian teratomas are the most frequent type of ovarian mass during childhood and adolescence [1]. The incidence of adnexal masses is up to 2.6 patients/100,000 children per year [2] and about 75% of them are of benign pathology [3].

The word “Teratomas” has its roots in Greek language, since it comes from Greek “terato”, which means monster, and the word “onkoma”, which means “mass or swelling” [4]. These tumours are embryonal neoplasms, where two or three germ layers are present [5]. Incidence, histology or clinical presentation is different in children and adolescents compared to those tumours in adults [5]. The process of the formation of teratomas is caused by the formation of germ layers such as ectoderm, endoderm and mesoderm [6]. According to WHO classification, these tumours are divided into mature or immature teratomas. The lasts have the potential of malignant transformation [6]. Another classification by Gonzales-Crussi defines teratomas as mature and immature, which are less frequent (45.5%) [6]. The most common teratomas in children are the mature cystic, which are also known as dermoid cysts [7]. Oosterhuis et al. separated the ovarian teratomas according to their chromosomal profile [8]. This group also supported that mature cystic teratomas are result of numeric abnormalities, such as extra X chromosome, 7, 12 or 15 [8]. At the same time, Cushing et al. [9] showed that 95% of teratomas have a normal karyotope. Saida et al. [10] showed that the MAGE gene family of tumour rejection antigens can be related to the pathogenesis of teratomas.

These tumours can be diagnosed incidentally, when young patients are examined or investigated for other issues, such as menstrual abnormalities or hydronephrosis [11]. Most of the time, the clinical and radiological characteristics or serum markers will support the diagnosis of such tumours [12]. Patients will be presented with abdominal or pelvic pain, associated with palpable pelvic mass [11]. In other cases, patients will suffer from acute sharp pain and potentially could be presented with acute abdomen and signs of peritonitis, because of ovarian torsion or haemorrhage [13].

There is no clear guidance for the management of ovarian teratomas in children and adolescents [14]. It is necessary, however, to understand the features of these tumours and the indications for operating on them and to put subjective criteria, which will help the management in elective and in emergency presentations. Spinelli et al. [13] showed that the sooner the diagnosis, the better for treatment and fertility preservation. Management options are oophorectomy or ovarian-sparing surgery (OSS). Surgical approaches include the use of minimally invasive surgery (MIS) in addition to an open approach [15].

This is a narrative review that aims to present the evidence on the presentation, diagnosis and management of ovarian teratomas in childhood and adolescence. The number of studies is small, and most of them are retrospective; in addition, there is heterogeneity that does not allow the performance of a systematic review.

## 2. Materials and Methods

We carried out a retrospective study, after the Institutional Ethics Committee and Review Board approval. We reviewed the medical records of patients between 12 and 20 years of age, who had surgery for ovarian teratomas between January 2000 and August 2022 in the Department of Obstetrics and Gynaecology in University Hospital of Ioannina. We collected relevant patient data, i.e., age at diagnosis and clinical presentation. In addition, we gathered information about imaging with trans-abdominal Ultrasonography (US), CT scans and/or Magnetic Resonance Imaging (MRI). These patients had also biochemical markers serum measured. Each patient was gathered based on clinical features (acute abdominal pain, chronic abdominal pain and palpable mass) and ultrasonographic appearance of mass (solid, complex and cystic). Surgeries performed include ovary-sparing surgery or oophorectomy, laparoscopic or open surgery.

PubMed database was searched to identify Reviews and Systematic Reviews published in the last 22 years (January 2000 to August 2022) using keywords such as: ovarian teratoma, paediatrics and adolescents.

## 3. Results

Forty patients were enrolled with a mean age at diagnosis of 11.8 years (12–18 years). The mean age of patients with mature teratomas was 12.17 years, while a mean age of 8.90 years was present in case of immature forms. Mature cystic teratomas accounted for 90% of all masses (n = 36/40), while 10% (n = 4/40) were immature teratomas. Malignant tumours were not recorded.

In 30% (n = 12/40) of our cases, acute abdominal pain due to adnexal torsion was the primary symptom, although in 70% (n = 28/40), it was chronic abdominal pain or abdominal/pelvic discomfort.

The ultrasonographic appearance of the neoplasms was complex in 96% (n = 38/40) and solid in the remaining 4% (n = 2/40) of cases. Serum levels of biochemical markers (AFP, HCG, Ca125, Ca19.9, CEA) were tested in all cases, according to clinical practice in our department. As result, they were found to be elevated in eight cases (20%).

All patients underwent surgery: 75% (n = 30/40) were oophorectomies and 25% (n = 10/40) were ovary-sparing surgeries. Laparoscopy was performed in 15% (n = 6/40) of the surgical interventions, while the remaining 85% (n = 34/40) were conducted via laparotomic access. Surgical notes mentioned that the decision for the type of operation was based on the size of the mass. In these 34 cases, the ovarian cysts were more than 8 cm in 60% of cases. Laparoscopy performed in 6 girls with mean age 14 years of age, with normal BMI (having a range of 22–28) and the size of ovarian mass was less than 6 cm. Two out of six patients (33.33%) had the mass retrieved intact from abdominal cavity, at the rest four cases, rupture of the mass took place and had to have extended washing and a course of antibiotics for seven days. In addition, the ovarian sparing surgeries performed in cases of mature ovarian teratomas, apart from one case, where histology came back as immature. As part of the follow up, these girls had regular ultrasound, where no evidence of recurrence noted for the following two years.

**Figure 1:**
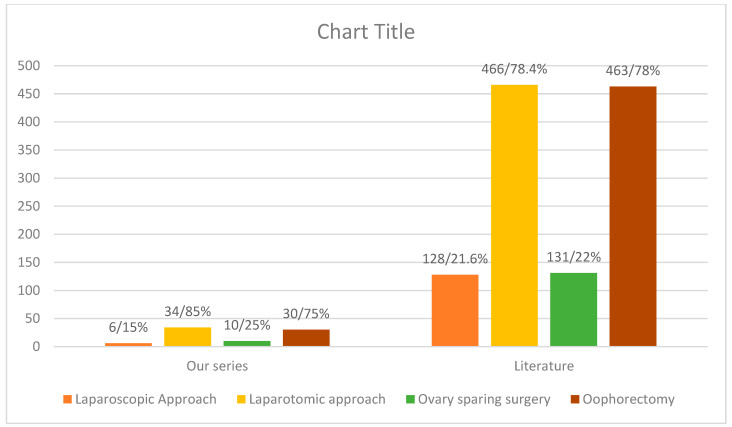
Surgical approach in ovarian teratomas over the years.

The literature search obtained 13 articles [1,14,16,17,18,19,20,21,22,23,24,25] on ovarian teratomas. In total, there were 1418 cases, with a mean age of 10.4 years at the times of diagnosis. The clinical presentation of these cases includes acute abdominal pain in 35.7% of cases (506/1418), chronic abdominal pain in 1.4% (192/1418), and a palpable mass in 1% (146/1418) (Table 1). The rest were asymptomatic, and it was an accidental finding. In total, 481 (34%) of girls suffered from adnexal torsion and only 57 had elevated tumour markers. From 1418 cases with ovarian teratomas, 594 patients underwent an operation (42%). A laparoscopic approach took place in 128 cases, while a laparotomic approach took place in 466 cases (78.4%) (Figure 1). We identified that 463 patients had an oophorectomy and 131 ovarian-sparing surgery (Figure 1). Histology confirmed the presence of mature teratomas in 1097 cases (77.4%), immature teratomas in 251 patients and only 2 mixed teratomas. From 2016 and onwards, an increased number was noticed of cases with an ovarian-sparing approach (from 0% in 2016 to 78% in 2018), along with a steady decreased rate of oophorectomies (from 100% in 2016 to 22% in 2018). The situation with the laparoscopic and open approaches during the last decade is similar.

## 4. Discussion

Pathogenesis, classification and treatment of teratomas are still debatable, even though the ovarian teratomas are quite common [11,26]. It is difficult to gather the information available in the literature. First of all, because of different histopathological terminology (immature teratoma, malignant teratoma and teratoma with malignant elements) and the existence of different staging systems [20]. In addition, research is focused on ovarian masses in general and not as much on ovarian teratomas. Most studies also analysed adult and paediatric patients together, without providing separate data for these different age groups [20].

Ovarian teratomas are more common in premenarchal age, between 11–12 years of age [13]. Zhang et al. [20] reported no difference in mean age of diagnosis between mature and immature teratomas. Their study included 521 ovarian masses, from which 289 were teratomas mature or immature, but without difference in mean age of diagnosis. Oltman et al. [26] showed that patients between the age of 1–8 years old had a higher incidence of malignant tumours (22%), when compared with older patients between the age of 15–19 years old. We revealed that the mean age at the time of diagnosis is 10.4 years old.

Patients with ovarian masses usually present without specific clinical signs or symptoms, and it can easily be confused with other benign conditions [26]. As a result, its differential diagnosis can be difficult or delayed. In case of ovarian torsion or rupture, patients are presented with abdominal or pelvic tenderness, nausea or vomiting, with or without fever [27,28]. Among the ovarian teratomas, mature cystic teratoma is related with adnexal torsion the most, while the malignant masses are less likely to be twisted because of the adhesions to surrounding structures, due to desmoplastic reactions [29,30]. Confirming these results, Spinelli et al. showed that 28 out of 31 cases with adnexal torsions referred to benign teratomas. Spinelli et al. reposted that 28 out of 31 cases with adnexal torsions referred to benign teratomas [31].

Evidence from the literature showed that tumour markers, such as AFP, B-hCG, CA125, CA19.9 and LDH, can be confusing. They can add information in differential diagnosis between benign and malignant germ cell tumour; nevertheless, 20% of paediatric benign ovarian neoplasms can be associated with increased levels of tumour markers [32]. Spinelli et al. observed that biological markers can be increased in some benign tumours in 20% of cases [32]. Loh et al. showed that AFP has a specificity of 88% and sensitivity of 50% for benign tumours [33]. The management of these tumours cannot be based only on the markers, as it is necessary to combine the clinical picture, imaging and histopathology.

The first line tool used for the investigation of ovarian mass is the ultrasound. One of the most widespread and accurate system of assessment of this pathology is the International Ovarian Tumor Analysis (IOTA). The IOTA system is based on characteristics that confirm the presence of the features of the 5M-rules or the 5 B-rules to classify an ovarian mass as benign, malignant or unclassifiable [34]. The best preoperative indicators to predict ovarian malignancy are thought to be the IOTA system rules, with an 8 cm cut-off and the heterogeneous features at Ultrasound or CT [26,27,35]. Renaud et al. reported that any solid components in ovarian mass suggests malignancy, but the cystic appearance of the mass has 100% sensitivity for benign forms [29]. The ovary crescent sign, which represents a ridge of ovarian tissue next to the mass, is a sign of benign mass with a specificity of 92–93% and sensibility of 96–100% [29]. There are some signs and features that can indicate the malignant or benign nature of an ovarian mass, although no single diagnostic examination is strong enough to provide a diagnosis. That is the reason why the combination of ultrasound, doppler ultrasound (especially for suspected adnexal torsion), CT scan and MRI is recommended, to obtain as much pre-operative information as possible and to plan the management of each case better [27].

Michelotti et al. reported that tumours addressed by laparoscopy had a median size of 6 cm and by laparotomy of 11.5 cm [15]. Braungart et al. compared the English reality with the Egyptian [36]. They showed that 55% and 62% of paediatric surgeons in the UK with and without an oncology interest, respectively, would perform MIS in benign-looking tumours with diameters between 3 and 10 cm, while 66% of all Egyptian surgeons would do the same [14,36]. Cystic lesions with a maximum size of 8–10 cm and negative markers are more likely to be predictive of benign masses [37]. Laparoscopic management of ovarian tumours is an accepted modality with well-known advantages; however, careful selection of patients is fundamental to avoid potential complications, such as rupture and spillage.

Elgedy et al. [14] proved that the ovarian-sparing methodology is related to MIS. The link between the laparoscopic approach and OSS has been previously reported already [1,38]. Technological improvements, such as a higher magnification of the endoscopic camera, better and more efficient types of energy and the ability to dissect small-sized lesions, can explain the above results [14]. Gonzalez et al. confirmed that children with emergency admissions were less likely to have OSS [39]. Rousseau et al., however, described that half of their patients were managed by OSS. The reason was the time of referral or diagnosis of torsion [40]. It is necessary to understand that in any girl presenting with acute abdomen pain, we ought to exclude ovarian torsion, since early detection can potentially save a patient’s ovary.

## 5. Conclusions

Ovarian-sparing surgery might be successfully applied in case of ovarian teratomas in children, but after triaging and selecting these children. Laparotomy is the treatment of choice in large masses, suspicious for malignancy, and if surgical staging is required. MIS is gaining increasingly more supporters, mainly because the skills of laparoscopic surgeons have increased dramatically in the last 20 years. Better-organised and -planned prospective multi-centre studies are necessary to obtain a deeper knowledge of the physiology and prognostic factors of teratomas in children.

## Figures and Tables

**Table 1:** Data from our series and from literature review.

Item	Our Series (n = 40)	Literature (n = 1418)
Mean age at diagnosis	11.8	10.4
Adnexal torsion	12	481
Biochemical markers elevated	8	57
Oophorectomy	30	463
Ovary-sparing surgery	10	131
Laparoscopic approach	6	128
Laparotomic approach	34	466
Acute abdominal pain	12	506
Chronic abdominal pain	27	192
Palpable mass	Not mentioned	146

## Data Availability

Data available on request due to restrictions of privacy.
